# Supplementary material for: Acrylamide Exposure and Cardiovascular Risk: A Systematic Review
Source: Nutrients. 2024 Dec 11;16(24):4279. doi: 10.3390/nu16244279 (PMC11677207; doi:10.3390/nu16244279)
Supplement: Supplementary file 1 [file nutrients-16-04279-s001.zip › nutrients-3341745-supplementary.pdf]

SUPPLEMENTARY MATERIALS

Acrylamide Exposure and Cardiovascular Risk: A Systematic Review

Diana María Mérida [1,2,3], Jimena Rey-García [4], Belén Moreno-Franco [5,6,7] and Pilar Guallar-Castillón [1,3,8,*]

Table S1. Studies excluded.

No.	Author (year)	Title	Reason of exclusion
1	Sobel W. et al. (1986) [1]	Acrylamide cohort mortality study	Duplicated of Swaen G.M. (2007)
2	Hogan K.A. et al. (1990) [2]	Mortality patterns and acrylamide exposure	No primary data: letter to the editor
3	Uzum G. et al. (1994) [3]	Alterations of brain microenvironment homeostasis in acrylamide neuropathy and effects of hypertension	Other outcomes: neuropathy
4	Collins J.J. et al. (1999) [4]	Mortality patterns among workers exposed to acrylamide	Duplicated of Marsh G.M. (2007)
5	Marsh G.M. (1999) [5]	Mortality patterns among workers exposed to acrylamide: 1994 follow up	Duplicated of Marsh G.M. (2007)
6	Dybing E. et al. (2003) [6]	Risk assessment of acrylamide in foods	Other outcomes: cancer
7	Naruszewicz M. et al. (2003) [7]	Trans-unsaturated fatty acids and acrylamide in food as potential atherosclerosis progression factors. Based on own studies	Other outcomes: oxidative stress
8	Shipp A. et al. (2006) [8]	Acrylamide: Review of toxicity data and dose-response analyses for cancer and noncancer effects	Other outcomes: reproductive effects and neurotoxicity
9	Parzefall W. et al. (2008) [9]	Minireview on the toxicity of dietary acrylamide	Review
10	Vesper H.W. et al. (2008) [10]	Cross-sectional study on acrylamide hemoglobin adducts in subpopulations from the European Prospective Investigation into Cancer and Nutrition (EPIC) Study	Different perspectives: acrylamide was analyzed as an outcome not as exposure
11	Gargas M.L. et al. (2009) [11]	Acrylamide: Consideration of species differences and nonlinear processes in estimating risk and safety for human ingestion	Animals
12	Naruszewicz M. et al. (2009) [12]	Chronic intake of potato chips in humans increases the production of reactive oxygen radicals by leukocytes and increases plasma C-reactive protein: a pilot study	Different exposure: potatoe chips intake
13	Camire M.E. et al. (2009) [13]	Potatoes and Human Health	Review

14	Tessier F.J.et al. (2012) [14]	Health effects of dietary Maillard reaction products: The results of ICARE and other studies	Review
15	Pingot D. et al. (2013) [15]	Toxicity of acrylamide and its metabolite - glicydamide	Review
16	Vesper H.W. et al. (2013) [16]	Among 10 sociodemographic and lifestyle variables, smoking is strongly associated with biomarkers of acrylamide exposure in a representative sample of the U.S. Population	Different perspectives: acrylamide was analyzed as an outcome not as exposure
17	Ng C.Y. et al. (2014) [17]	Heated vegetable oils and cardiovascular disease risk factors	Review
18	Sellier C. et al. (2015) [18]	Acrylamide induces accelerated endothelial aging in a human cell model	Other outcomes: endothelial aging
19	Campbell L.R. et al. (2015) [19]	Study of cardiovascular disease biomarkers among tobacco consumers, part 1: Biomarkers of exposure	Different exposure: tobacco biomarkers
20	De Long N.E. et al. (2017) [20]	Early-life chemical exposures and risk of metabolic syndrome	Review
21	Kadawathagedara M. et al. (2018) [21]	Dietary acrylamide intake during pregnancy and postnatal growth and obesity: Results from the Norwegian Mother and Child Cohort Study (MoBa)	Pregnant women
22	Furrer A.N. et al. (2018) [22]	Impact of potato processing on nutrients, phytochemicals, and human health	Review
23	Keith R.J. et al. (2018) [23]	Protocol to assess the impact of tobacco-induced volatile organic compounds on cardiovascular risk in a cross-sectional cohort: Cardiovascular Injury due to Tobacco Use study	Not primary data: protocol
24	Gupta U.C. et al. (2019) [24]	The Important Role of Potatoes, An Underrated Vegetable Food Crop in Human Health and Nutrition	Review
25	Shrivastava, S. et al. (2019) [25]	Protective efficacy of vitamin F against acrylamide induced toxicity: Studies on oxidative stress biomarkers	Animals
26	Nematollahi A. et al. (2020) [26]	Acrylamide content of collected food products from Tehran's market: a risk assessment study	Other outcomes: lifetime cancer risk

27	Egusquiza R.J. et al. (2020) [27]	Environmental obesogens and their impact on susceptibility to obesity: New mechanisms and chemicals	Review
28	Tardiff R.G. et al. (2020) [28]	Estimation of safe dietary intake levels of acrylamide for humans	Other outcomes: neurotoxicity and carcinogenicity
29	Wang S.Y. et al. (2020) [29]	A urinary metabolomic study from subjects after long-term occupational exposure to low concentration acrylamide using UPLC-QTOF/MS	Different scope
30	Hsu C.N. et al. (2020) [30]	Association between Acrylamide Metabolites and Cardiovascular Risk in Children With Early Stages of Chronic Kidney Disease	No association estimates
31	Mielech A. et al. (2021) [31]	Assessment of the risk of contamination of food for infants and toddlers	Review
32	McGraw K.E. et al. (2021) [32]	Exposure to volatile organic compounds – acrolein, 1,3-butadiene, and crotonaldehyde – is associated with vascular dysfunction	Different exposure: acrolein, 1,3-butadiene, and crotonaldehyde
33	Amato A.A. et al. (2021) [33]	Obesity and endocrine-disrupting chemicals	Review
34	Hemgesberg M. et al. (2021) [34]	Acrylamide-derived DNA adducts in human peripheral blood mononuclear cell DNA: Correlation with body mass	No association estimates
35	Liu Z. et al. (2021) [35]	Associations of acrylamide with non-alcoholic fatty liver disease in American adults: a nationwide cross-sectional study	Other outcomes: non-alcoholic fatty liver
36	Shi K. et al. (2022) [36]	Biscuit consumption and diabetic retinopathy incidence in adults in the United States	Different exposure: biscuit intake
37	Wan X. et al. (2022) [37]	Machine learning prediction of exposure to acrylamide based on modelling of association between dietary exposure and internal biomarkers	Other associacions
38	Wan X. et al. (2022) [38]	Metabolomics strategy comprehensively unveils the effect of catechins intervention on the biomarkers of exposure to acrylamide and biomarkers of cardiometabolic risk	Animals
39	Marković Filipović J. et al. (2022) [39]	Acrylamide and Potential Risk of Diabetes Mellitus: Effects on Human Population, Glucose Metabolism and Beta-Cell Toxicity	Review

40	Guth S. et al. (2023) [40]	Evaluation of the genotoxic potential of acrylamide: Arguments for the derivation of a tolerable daily intake (TDI value)	Other outcomes: genotoxicity
41	Buyukdere Y. et al. (2023) [41]	From a toxin to an obesogen: a review of potential obesogenic roles of acrylamide with a mechanistic approach	Review
42	Steele E.M. et al. (2023) [42]	Ultra-processed food consumption and exposure to acrylamide in a nationally representative sample of the US population aged 6 years and older	Other associacions
43	Govindaraju, I. et al. (2024) [43]	Dietary Acrylamide: A Detailed Review on Formation, Detection, Mitigation, and Its Health Impacts	Review
44	Mendy, A. et al. (2024) [44]	Urinary Volatile Organic Compound Metabolites Are Associated with Reduced Lung Function in U.S. Children and Adolescents	Other outcomes: lung function

Table S2. Risk of bias of the studies according to the Joanna Briggs Institute critical appraisal tools.

**Author (year)**	**Q1**	**Q2**	**Q3**	**Q4**	**Q5**	**Q6**	**Q7**	**Q8**	**Q9**	**Q10**	**Q11**	**Yes (%)**	**Overall risk of bias**
**CVD mortality**													
Marsh G.M. et al (2007)	Y	Y	N	Y	U	U	Y	Y	U	U	Y	54.5	Moderate
Swaen G.M. et al. (2007)	N	Y	N	Y	U	U	Y	Y	U	U	Y	45.4	High
Huang M. et al. (2018)	Y	Y	Y	Y	Y	Y	Y	U	U	U	Y	72.7	Low
Wu H. et al. (2022)	Y	Y	U	Y	Y	Y	Y	N	U	U	Y	63.6	Moderate
Marques C. et al. (2023)	Y	Y	N	Y	Y	U	Y	Y	U	U	Y	63.6	Moderate
Feng X. et al. (2024)	Y	Y	Y	Y	Y	Y	Y	Y	U	U	Y	81.8	Low
Nalini M.et al. (2024)	Y	Y	Y	Y	Y	Y	Y	Y	U	U	Y	81.8	Low
**CVD risk**													
Zhang Y. et al. (2018)	Y	Y	Y	Y	Y	Y	N	Y				87.5	Low
Wang B. et al. (2022)	Y	Y	Y	Y	Y	Y	N	Y				87.5	Low
Wang X. et al. (2023)	Y	Y	Y	Y	Y	Y	N	Y				87.5	Low
Han S. et al. (2024)	Y	Y	Y	Y	Y	Y	N	Y				87.5	Low
Ma M. et al. (2023)	Y	Y	Y	Y	Y	Y	N	Y				87.5	Low
**Diabetes and glucose metabolism**													
Lin C-Y. et al. (2009)	Y	Y	U	Y	Y	Y	Y	Y				87.5	Low
Lin C-Y. et al. (2013)	Y	Y	Y	Y	Y	Y	Y	Y				100	Low
Wang B. et al. (2020)	Y	Y	Y	Y	Y	Y	Y	Y				100	Low
Yin G. et al. (2021)	Y	Y	Y	Y	Y	Y	Y	Y				100	Low
Hosseini-Esfahani F. et al. (2023)	Y	Y	N	Y	Y	Y	Y	N	U	U	Y	63.6	Moderate
**Dyslipidemia and lipid metabolism**													
Cheang I. et al. (2020)	Y	Y	Y	Y	Y	Y	Y	Y				100	Low
Chen W.Y. et al. (2023)	Y	Y	Y	Y	Y	Y	Y	Y				100	Low
**Obesity and body composition**													
Chu P-L. et al. (2017)	Y	Y	Y	Y	Y	Y	Y	Y				100	Low
Huang M. et al. (2018)	Y	Y	Y	Y	Y	Y	Y	Y				100	Low
Yin T. et al. (2022)	Y	Y	Y	Y	Y	Y	Y	Y				100	Low
Lei T. et al. (2023)	Y	Y	Y	Y	Y	Y	Y	Y				100	Low

**Hypertension and blood pressure**												
Liang J. et al. (2022)	Y	Y	U	Y	Y	Y	Y	Y				87.5	Low
McGraw K.E. et al. (2023)	Y	Y	U	Y	Y	Y	Y	Y				87.5	Low
**Metabolic syndrome**												
Hung C-C. et al. (2021)	U	Y	U	Y	Y	Y	Y	Y				75	Low
Wan X. et al. (2022)	Y	Y	Y	Y	Y	Y	Y	Y				100	Low
Tan L. et al. (2024)	Y	Y	Y	Y	Y	Y	Y	Y				100	Low

Y= yes, N= No, U= unclear; NE: not evaluated.

Cross-sectional studies:
Q1. Were the criteria for inclusion in the sample clearly defined?
Q2. Were the study subjects and the setting described in detail?
Q3. Was the exposure measured in a valid and reliable way?
Q4. Were objective, standard criteria used for measurement of the condition?
Q5. Were confounding factors identified?
Q6. Were strategies to deal with confounding factors stated?
Q7. Were the outcomes measured in a valid and reliable way?
Q8. Was appropriate statistical analysis used?

Cohort studies:
Q1. Were the two groups similar and recruited from the same population?
Q2. Were the exposures measured similarly to assign people to both exposed and unexposed groups?
Q3. Was the exposure measured in a valid and reliable way?
Q4. Were confounding factors identified?
Q5. Were strategies to deal with confounding factors stated?
Q6. Were the groups/participants free of the outcome at the start of the study?
Q7. Were the outcomes measured in a valid and reliable way?
Q8. Was the follow up reported and sufficient to be long enough for outcomes to occur?
Q9. Was follow-up complete, and if not, were the reasons to loss to follow up described and explored?
Q10. Were strategies to address incomplete follow up?
Q11. Was appropriate statistical analysis used?

Table S3. Association between acrylamide exposure and cardiovascular risk factors in non-smokers.

Study	Country (sample)	Acrylamide metabolite	Unit of comparison	Outcome								Results
				CVD mortality	IHD mortality	CVD	10-y CVD risk	DM	GO	HTN	MetS	
Huang M. (2018)	US (NHANES 2003-2006)	HbAA	Q4vsQ1	NSA								CVD mortality: HR= 1.54 (0.35-6.76), p-trend: 0.648
		HbGA	Q4vsQ1	↓								CVD mortality: HR= 0.22 (0.07-0.71), p-trend: 0.001
		HbAA+HbGA	Q4vsQ1	NSA								CVD mortality: HR= 0.36 (0.11-1.14), p-trend: 0.121
		HbGA/HbAA	Q4vsQ1	↓								CVD mortality: HR= 0.12 (0.03-0.45), p-trend: 0.004
Nalini M.et al. (2024)	Iran (Golestan Cohort Study)	AAMA	T3vsT1		↑							IHD mortality: HR= 2.02 (1.19-3.44)
		GAMA	T3vsT1		NSA							IHD mortality: HR= 1.18 (0.66-2.12)
Zhang Y. (2018)	US (NHANES 2003-2006)	HbAA	Q4vsQ1			NSA						CVD: OR= 0.72 (0.21-2.50), p-trend: 0.590
		HbGA	Q4vsQ1			NSA						CVD: OR= 0.43 (0.09-2.06), p-trend: 0.155
		HbAA+HbGA	Q4vsQ1			NSA						CVD: OR= 0.35 (0.11-1.10), p-trend: 0.065
		HbGA/HbAA	Q4vsQ1			NSA						CVD: OR= 0.39 (0.12-1.32), p-trend: 0.165
Huang M. (2018)	US (NHANES 2003-2006)	HbGA/HbAA	Q4vsQ1						↑			GO: OR= 3.00 (2.44-3.70), p-trend: <0.0001
Yin G. (2021)		HbAA	Q4vsQ1					↓				DM: OR= 0.46 (0.24-0.89), p-trend: 0.177

	US (NHANES 2005-2006, 2013-2016)	HbGA/HbAA	Q4vsQ1					↑				DM: OR= 1.55 (1.03-2.34), p-trend: 0.042
Wan X. (2022)	US (NHANES 2003-2006, 2013-2016)	HbGA/HbAA	1-unit increase in natural log HbGA/HbAA								↑	MetS: OR= 1.76 (1.22-2.56), p-trend: ND
Wang B. (2022)	China (Wuhan-Zhuhai cohort)	AAMA	Q4vsQ1			↑						10-y CVD risk: OR= 1.50 (1.10-2.04), p-trend: 0.040
		GAMA	Q4vsQ1				↑					10-y CVD risk: OR= 1.73 (1.27-2.35), p-trend: <0.001
		ΣUAAM	Q4vsQ1				↑					10-y CVD risk: OR= 1.55 (1.14-2.11), p-trend: 0.009
		GAMA/AAMA	Q4vsQ1			NSA						10-y CVD risk: OR= 1.23 (0.90-1.69), p-trend: 0.080
Hosseini-Esfahani F. (2023)	Iran (Tehran lipid and glucose study)	Acrylamide intake	Q4vsQ1					NSA				DM: HR= 1.09 (0.98-1.22), p-trend: 0.24
McGraw K.E. (2023)	US (Jackson Heart Study cohort)	AAMA	Per IQR of AAMA							NSA		HTN: RR= 1.00 (0.97-1.02), p-value: 0.88

AAMA: N-acetyl-S-(2-carbamoylethyl)-l-cysteine); CVD: cardiovascular disease; DM: diabetes mellitus; GAMA: N-acetyl-S-(2-carbamoyl-2-hydroxyethyl)-l-cysteine; GO: general obesity; HbAA: hemoglobin adducts of acrylamide; HbGA: hemoglobin adducts of glycidamide; IHD: ischemic heart disease; MetS: metabolic syndrome; NSA: not significant association; OR: odds ratio; RR: relative risk; US: United States.

Table S4. Association between acrylamide exposure and cardiovascular risk factors in smokers.

Study	Country (sample)	Acrylamide metabolite	Unit of comparison	Outcome						Results
				IHD mortality	CVD	DM	GO	HTN	MetS	
Nalini M.et al. (2024)	Iran (Golestan Cohort Study)	AAMA	T3vsT1	NSA						IHD mortality: HR= 0.41 (0.16-1.06)
		GAMA	T3vsT1	NSA						IHD mortality: HR= 0.95 (0.51-1.76)
Zhang Y. (2018)	US (NHANES 2003-2006)	HbAA	Q4vsQ1		↑					CVD: OR= 3.67 (1.49-9.05), p-trend: 0.009
		HbGA	Q4vsQ1		NSA					CVD: OR= 0.44 (0.18-1.06), p-trend: 0.106
		HbAA+HbGA	Q4vsQ1		NSA					CVD: OR= 1.67 (0.97-2.87), p-trend: 0.068
		HbGA/HbAA	Q4vsQ1		↓					CVD: OR= 0.41 (0.20-0.84), p-trend: 0.014
Huang M. (2018)	US (NHANES 2003-2006)	HbGA/HbAA	Q4vsQ1				↑			GO: OR= 2.49 (1.99-3.11), p-trend: <0.0001
Yin G. (2021)	US (NHANES 2005-2006, 2013-2016)	HbAA	Q4vsQ1			↓				DM: OR= 0.67 (0.47-0.94), p-trend: 0.035
		HbGA/HbAA	Q4vsQ1			↑				DM: OR= 2.32 (1.64-3.28), p-trend: <0.001
Wan X. (2022)	US (NHANES 2003-2006, 2013-2016)	HbGA/HbAA	1-unit increase in natural log HbGA/HbAA						NSA	MetS: OR= 1.30 (0.84-2.01), p-trend: ND
Hosseini-Esfahani F. (2023)	Iran (Tehran lipid and glucose study)	Acrylamide intake	Q4vsQ1			NSA				DM: HR= 1.14 (0.94-1.38), p-trend: 0.18
McGraw K.E. (2023)	US (Jackson Heart Study cohort)	AAMA	Per IQR of AAMA					NSA		HTN: RR= 1.02 (0.91-1.13), p-value: 0.78

AAMA: N-acetyl-S-(2-carbamoylethyl)-l-cysteine); CVD: cardiovascular disease; DM: diabetes mellitus; GAMA: N-acetyl-S-(2-carbamoyl-2-hydroxyethyl)-l-cysteine; GO: general obesity; HbAA: hemoglobin adducts of acrylamide; HbGA: hemoglobin adducts of glycidamide; IHD: ischemic heart disease; MetS: metabolic syndrome; NSA: not significant association; OR: odds ratio; RR: relative risk; US: United States.

REFERENCES

1. Sobel W, Bond GG, Parsons TW, Brenner FE. Acrylamide cohort mortality study. Br J Ind Med [Internet]. 1986 [cited 2024 Aug 2];43:785–8. Available from: https://www.ncbi.nlm.nih.gov/pmc/articles/pmid/3790460/?tool=EBI

2. Hogan KA, Scott CLS. Mortality patterns and acrylamide exposure. J Occup Med [Internet]. 1990 [cited 2024 Aug 2];32:947. Available from: https://pubmed.ncbi.nlm.nih.gov/2074523/

3. Uzum G, Diler S, Curgunlu S, Hacialioglu M, Ercan S, Ziylan YZ. Alterations of brain microenvironment homeostasis in acrylamide neuropathy and effects of hypertension. Istanbul Tip Fak Mecmuasi. 1994;57:9–12.

4. Collins JJ, Swaen GMH, Marsh GM, Utidjian M, Caporossi JC, Lucas LJ. Mortality patterns among workers exposed to acrylamide. J Occup Med [Internet]. 1989 [cited 2024 Aug 2];31:614–7. Available from: https://pubmed.ncbi.nlm.nih.gov/2769457/

5. Marsh GM, Lucas LJ, Youk AO, Schall LC. Mortality patterns among workers exposed to acrylamide: 1994 follow up. Occup Environ Med [Internet]. 1999 [cited 2024 Aug 2];56:181–90. Available from: https://pubmed.ncbi.nlm.nih.gov/10448327/

6. Dybing E, Sanner T. Risk assessment of acrylamide in foods. Toxicol Sci [Internet]. 2003 [cited 2024 Aug 2];75:7–15. Available from: https://pubmed.ncbi.nlm.nih.gov/12805639/

7. Naruszewicz M, Daniewski M, Nowicka G, Kozłowska-Wojciechowska M. Trans-unsaturated fatty acids and acrylamide in food as potential atherosclerosis progression factors. Based on own studies. Acta Microbiol Pol [Internet]. 2003 [cited 2024 Aug 2];52 Suppl:75–81. Available from: https://pubmed.ncbi.nlm.nih.gov/15058816/

8. Shipp A, Lawrence G, Gentry R, McDonald T, Bartow H, Bounds J, et al. Acrylamide: review of toxicity data and dose-response analyses for cancer and noncancer effects. Crit Rev Toxicol [Internet]. 2006 [cited 2024 Aug 2];36:481–608. Available from: https://pubmed.ncbi.nlm.nih.gov/16973444/

9. Parzefall W. Minireview on the toxicity of dietary acrylamide. Food Chem Toxicol [Internet]. 2008 [cited 2024 Aug 2];46:1360–4. Available from: https://pubmed.ncbi.nlm.nih.gov/17905504/

10. Vesper HW, Slimani N, Hallmans G, Tjønneland A, Agudo A, Benetou V, et al. Cross-sectional study on acrylamide hemoglobin adducts in subpopulations from the European Prospective Investigation into Cancer and Nutrition (EPIC) Study. J Agric Food Chem [Internet]. 2008 [cited 2024 Aug 2];56:6046–53. Available from: https://pubmed.ncbi.nlm.nih.gov/18624432/

11. Gargas ML, Kirman CR, Sweeney LM, Tardiff RG. Acrylamide: Consideration of species differences and nonlinear processes in estimating risk and safety for human ingestion. Food Chem Toxicol [Internet]. 2009 [cited 2024 Aug 2];47:760–8. Available from: https://pubmed.ncbi.nlm.nih.gov/19166901/

12. Naruszewicz M, Zapolska-Downar D, Kośmider A, Nowicka G, Kozłowska-Wojciechowska M, Vikström AS, et al. Chronic intake of potato chips in humans increases the production of reactive oxygen radicals by leukocytes and increases plasma C-reactive protein: a pilot study. Am J Clin Nutr [Internet]. 2009 [cited 2024 Aug 2];89:773–7. Available from: https://pubmed.ncbi.nlm.nih.gov/19158207/

13. Camire ME, Kubow S, Donnelly DJ. Potatoes and human health. Crit Rev Food Sci Nutr [Internet]. 2009 [cited 2024 Aug 2];49:823–40. Available from: https://pubmed.ncbi.nlm.nih.gov/19960391/

14. Tessier FJ, Birlouez-Aragon I. Health effects of dietary Maillard reaction products: the results of ICARE and other studies. Amino Acids [Internet]. 2012 [cited 2024 Aug 2];42:1119–31. Available from: https://pubmed.ncbi.nlm.nih.gov/20949364/

15. Pingot D, Pyrzanowski K, Michałowicz J, Bukowska B, Łódzki U, Biofizyki K, et al. Toksyczność akrylamidu i jego metabolitu – glicydamidu. Medycyna Pracy Workers' Health and Safety [Internet]. 2014 [cited 2024 Aug 2];64:259–71. Available from: https://medpr.imp.lodz.pl/Toksycznosc-akrylamidu-i-jego-metabolitu-glicydamidu,444,0,1.html

16. Vesper HW, Sternberg MR, Frame T, Pfeiffer CM. Among 10 sociodemographic and lifestyle variables, smoking is strongly associated with biomarkers of acrylamide exposure in a representative sample of the U.S. Population. J Nutr [Internet]. 2013 [cited 2024 Aug 2];143. Available from: https://pubmed.ncbi.nlm.nih.gov/23596166/

17. Ng CY, Leong XF, Masbah N, Adam SK, Kamisah Y, Jaarin K. Heated vegetable oils and cardiovascular disease risk factors. Vascul Pharmacol [Internet]. 2014 [cited 2024 Aug 2];61:1–9. Available from: https://pubmed.ncbi.nlm.nih.gov/24632108/

18. Sellier C, Boulanger E, Maladry F, Tessier FJ, Lorenzi R, Nevière R, et al. Acrylamide induces accelerated endothelial aging in a human cell model. Food Chem Toxicol [Internet]. 2015 [cited 2024 Aug 2];83:140–5. Available from: https://pubmed.ncbi.nlm.nih.gov/26070502/

19. Campbell LR, Brown BG, Jones BA, Marano KM, Borgerding MF. Study of cardiovascular disease biomarkers among tobacco consumers, part 1: biomarkers of exposure. Inhal Toxicol [Internet]. 2015 [cited 2024 Aug 2];27:149–56. Available from: https://pubmed.ncbi.nlm.nih.gov/25787703/

20. De Long NE, Holloway AC. Early-life chemical exposures and risk of metabolic syndrome. Diabetes Metab Syndr Obes [Internet]. 2017 [cited 2024 Aug 2];10:101. Available from: /pmc/articles/PMC5370400/

21. Kadawathagedara M, Botton J, de Lauzon-Guillain B, Meltzer HM, Alexander J, Brantsaeter AL, et al. Dietary acrylamide intake during pregnancy and postnatal growth and obesity: Results from the Norwegian Mother and Child Cohort Study (MoBa). Environ Int [Internet]. 2018 [cited 2024 Aug 2];113:325–34. Available from: https://pubmed.ncbi.nlm.nih.gov/29398013/

22. Furrer AN, Chegeni M, Ferruzzi MG. Impact of potato processing on nutrients, phytochemicals, and human health. Crit Rev Food Sci Nutr [Internet]. 2018 [cited 2024 Aug 2];58:146–68. Available from: https://pubmed.ncbi.nlm.nih.gov/26852789/

23. Keith RJ, Fetterman JL, Riggs DW, O'Toole T, Nystoriak JL, Holbrook M, et al. Protocol to assess the impact of tobacco-induced volatile organic compounds on cardiovascular risk in a cross- sectional cohort: Cardiovascular Injury due to Tobacco Use study. BMJ Open [Internet]. 2018 [cited 2024 Aug 2];8:19850. Available from: /pmc/articles/PMC5884372/

24. Gupta UC, Gupta SC. The Important Role of Potatoes, An Underrated Vegetable Food Crop in Human Health and Nutrition. Curr Nutr Food Sci [Internet]. 2018 [cited 2024 Aug 2];15:11–9. Available from: https://www.eurekaselect.com/article/92861

25. Shrivastava S, Nirala SK, Reshi MS, Shukla S, Sharma A, Uthra C. Protective Efficacy of Vitamin F against Acrylamide Induced Toxicity: Studies on Oxidative Stress Biomarkers. Open Biomark J. 2019;9:62–9.

26. Nematollahi A, Kamankesh M, Hosseini H, Ghasemi J, Hosseini-Esfahani F, Mohammadi A, et al. Acrylamide content of collected food products from Tehran's market: a risk assessment study. Environ Sci Pollut Res Int [Internet]. 2020 [cited 2024 Aug 2];27:30558–70. Available from: https://pubmed.ncbi.nlm.nih.gov/32468359/

27. Egusquiza RJ, Blumberg B. Environmental Obesogens and Their Impact on Susceptibility to Obesity: New Mechanisms and Chemicals. Endocrinology [Internet]. 2020 [cited 2024 Aug 2];161. Available from: https://pubmed.ncbi.nlm.nih.gov/32067051/

28. Tardiff RG, Gargas ML, Kirman CR, Leigh Carson M, Sweeney LM. Estimation of safe dietary intake levels of acrylamide for humans. Food Chem Toxicol [Internet]. 2010 [cited 2024 Aug 2];48:658–67. Available from: https://pubmed.ncbi.nlm.nih.gov/19948203/

29. Wang SY, Han D, Pan YL, Yu CP, Zhou XR, Xin R, et al. A urinary metabolomic study from subjects after long-term occupational exposure to low concentration acrylamide using UPLC-QTOF/MS. Arch Biochem Biophys. 2020;681:108279.

30. Hsu CN, Hou CY, Lu PC, Chang-Chien GP, Lin S, Tain YL. Association between Acrylamide Metabolites and Cardiovascular Risk in Children With Early Stages of Chronic Kidney Disease. Int J Mol Sci [Internet]. 2020 [cited 2024 Aug 2];21:1–12. Available from: /pmc/articles/PMC7461542/

31. Mielech A, Puścion-jakubik A, Socha K. Assessment of the Risk of Contamination of Food for Infants and Toddlers. Nutrients [Internet]. 2021 [cited 2024 Aug 2];13. Available from: /pmc/articles/PMC8308760/

32. McGraw KE, Riggs DW, Rai S, Navas-Acien A, Xie Z, Lorkiewicz P, et al. Exposure to volatile organic compounds - acrolein, 1,3-butadiene, and crotonaldehyde - is associated with vascular dysfunction. Environ Res [Internet]. 2021 [cited 2024 Aug 2];196. Available from: https://pubmed.ncbi.nlm.nih.gov/33636185/

33. Amato AA, Wheeler HB, Blumberg B. Obesity and endocrine-disrupting chemicals. Endocr Connect [Internet]. 2021 [cited 2024 Aug 2];10:R87–105. Available from: https://pubmed.ncbi.nlm.nih.gov/33449914/

34. Hemgesberg M, Stegmüller S, Cartus A, Hemmer S, Püttmann M, Stockis JP, et al. Acrylamide-derived DNA adducts in human peripheral blood mononuclear cell DNA: Correlation with body mass. Food Chem Toxicol [Internet]. 2021 [cited 2024 Aug 2];157. Available from: https://pubmed.ncbi.nlm.nih.gov/34560178/

35. Liu Z, Wang J, Chen S, Xu C, Zhang Y. Associations of acrylamide with non-alcoholic fatty liver disease in American adults: a nationwide cross-sectional study. Environ Health [Internet]. 2021 [cited 2024 Aug 2];20. Available from: https://pubmed.ncbi.nlm.nih.gov/34461916/

36. Shi K, Chen Y, Zhu X, Wu J, Chen J, Hu J, et al. Biscuit consumption and diabetic retinopathy incidence in adults in the United States. Diabetol Metab Syndr [Internet]. 2022 [cited 2024 Aug 2];14. Available from: /pmc/articles/PMC9258145/

37. Wan X, Zhang Y, Gao S, Shen X, Jia W, Pan X, et al. Machine learning prediction of exposure to acrylamide based on modelling of association between dietary exposure and internal biomarkers. Food and Chemical Toxicology. 2022;170:113498.

38. Wan X, Jia W, Wang Q, Chen X, Wang A, Zhu L, et al. Metabolomics strategy comprehensively unveils the effect of catechins intervention on the biomarkers of exposure to acrylamide and biomarkers of cardiometabolic risk. Environ Int. 2022;169:107517.

39. Filipović JM, Karan J, Ivelja I, Matavulj M, Stošić M. Acrylamide and Potential Risk of Diabetes Mellitus: Effects on Human Population, Glucose Metabolism and Beta-Cell Toxicity. Int J Mol Sci [Internet]. 2022 [cited 2024 Aug 2];23. Available from: /pmc/articles/PMC9181725/

40. Guth S, Baum M, Cartus AT, Diel P, Engel KH, Engeli B, et al. Evaluation of the genotoxic potential of acrylamide: Arguments for the derivation of a tolerable daily intake (TDI value). Food and Chemical Toxicology. 2023;173:113632.

41. Buyukdere Y, Akyol A. From a toxin to an obesogen: a review of potential obesogenic roles of acrylamide with a mechanistic approach. Nutr Rev [Internet]. 2023 [cited 2024 Aug 2];82:128–42. Available from: https://dx.doi.org/10.1093/nutrit/nuad041

42. Martínez Steele E, Buckley JP, Monteiro CA. Ultra-processed food consumption and exposure to acrylamide in a nationally representative sample of the US population aged 6 years and older. Prev Med (Baltim) [Internet]. 2023 [cited 2024 Aug 2];174. Available from: https://pubmed.ncbi.nlm.nih.gov/37391037/

43. Govindaraju I, Sana M, Chakraborty I, Rahman MH, Biswas R, Mazumder N. Dietary Acrylamide: A Detailed Review on Formation, Detection, Mitigation, and Its Health Impacts. Foods [Internet]. 2024 [cited 2024 Aug 2];13. Available from: https://pubmed.ncbi.nlm.nih.gov/38397533/

44. Mendy A, Burcham S, Merianos AL, Mersha TB, Yolton K, Chen A, et al. Urinary Volatile Organic Compound Metabolites Are Associated with Reduced Lung Function in U.S. Children and Adolescents. Toxics [Internet]. 2024 [cited 2024 Aug 2];12:289. Available from: https://www.mdpi.com/2305-6304/12/4/289/htm
